# Tumor-associated neutrophils and macrophages interaction contributes to intrahepatic cholangiocarcinoma progression by activating STAT3

**DOI:** 10.1136/jitc-2020-001946

**Published:** 2021-03-10

**Authors:** Zhengjun Zhou, Pengcheng Wang, Rongqi Sun, Jia Li, Zhiqiang Hu, Haoyang Xin, Chubin Luo, Jian Zhou, Jia Fan, Shaolai Zhou

**Affiliations:** Department of Liver Surgery and Transplantation, Liver Cancer Institute, Zhongshan Hospital, Fudan University; Key Laboratory of Carcinogenesis and Cancer Invasion (Fudan University), Ministry of Education, Shanghai, China

**Keywords:** liver neoplasms, macrophages, neutrophil infiltration, tumor microenvironment, cytokines

## Abstract

**Background:**

Tumor-associated neutrophils (TANs) and macrophages (TAMs) can each influence cancer growth and metastasis, but their combined effects in intrahepatic cholangiocarcinoma (ICC) remain unclear.

**Methods:**

We explored the distributions of TANs and TAMs in patient-derived ICC samples by multiplex immunofluorescent staining and tested their separate and combined effects on ICC in vitro and in vivo. We then investigated the mechanistic basis of the effects using PCR array, western blot analysis and ELISA experiments. Finally, we validated our results in a tissue microarray composed of primary tumor tissues from 359 patients with ICC.

**Results:**

The spatial distributions of TANs and TAMs were correlated with each other in patient-derived ICC samples. Interaction between TANs and TAMs enhanced the proliferation and invasion abilities of ICC cells in vitro and tumor progression in a mouse xenograft model of ICC. TANs and TAMs produced higher levels of oncostatin M and interleukin-11, respectively, in co-culture than in monoculture. Both of those cytokines activated STAT3 signaling in ICC cells. Knockdown of STAT3 abolished the protumor effect of TANs and TAMs on ICC. In tumor samples from patients with ICC, increased TAN and TAM levels were correlated with elevated p-STAT3 expression. All three of those factors were independent predictors of patient outcomes.

**Conclusions:**

TANs and TAMs interact to promote ICC progression by activating STAT3.

## Introduction

Immune cells in the tumor microenvironment play important roles in cancer development.^1 2^ Immune cells can produce an antitumor immune response and directly kill malignant cells, but they can also be recruited and retrained by tumor cells to facilitate tumor growth and progression.^3 4^ As the pathogenic role of the immune response, and particularly the inflammatory response, in cancer development becomes increasingly clear, chronic inflammation is now recognized as a hallmark of cancer.^5^ Tumor-associated macrophages (TAMs) are present the tumor microenvironment of many cancers, where they play a key role in tumor initiation and progression by activating and maintaining cancer-related inflammation.^1 6^ In addition, there is increasing evidence that tumor-associated neutrophils (TANs) support tumorigenesis by promoting oncogenic transformation and tumor progression.^7–9^ However, other reports have shown that under certain conditions, such as those developed by therapeutic treatments, they can mediate antitumorous functions.^10^

Intrahepatic cholangiocarcinoma (ICC) is a cancer of the bile ducts that accounts for around 10%–15% of all primary hepatic malignancies.^11^ Chronic inflammation of the biliary epithelium is thought to play a central role in ICC carcinogenesis.^12 13^ The most common risk factor for ICC is primary sclerosing cholangitis, or inflammation and scarring of the bile ducts.^14 15^ Other risk factors for ICC include gallstones in the biliary ducts or common bile duct, Caroli’s disease, liver fluke infestation and infection with hepatitis C virus,^14 15^ all of which are associated with chronic inflammation.^16^ ICC tumors are characterized by a dense layer of connective tissue infiltrated by immune/inflammatory cells.^13^ During ICC progression, neoplastic cells communicate in a reciprocal manner with the surrounding stromal and immune cells,^2 13^ suggesting a functional relationship between chronic inflammation and ICC development.

To gain a better understanding of the functions and potential interactions of TANs and TAMs in ICC, we examined the spatial distributions of TANs and TAMs in ICC samples from human patients. Then, we investigated the separate and combined effects and mechanism of TANs and TAMs on ICC growth and metastasis in cell-culture experiments and in a mouse xenograft model of ICC. Finally, we analyzed the relationships between TAN and TAM levels and prognosis in patients with ICC.

## Materials and methods

### Patients and follow-up

For TAN and TAM isolation and PCR array analysis of target gene expression, we collected tumor tissues from 15 patients with ICC that underwent curative resection between March 2018 and April 2018 at the Liver Surgery Department, Zhongshan Hospital, Fudan University. To further examine cytokine expression by TANs and TAMs and in vitro functional assays and in vivo animal studies, we collected another set of tumor tissues from 30 patients with ICC that underwent curative resection between May 2018 and August 2018 in the same department.

For multiplex immunofluorescent staining, immunohistochemical and prognostic analyses, we enrolled two independent patient cohorts (Formalin-fixed, paraffin-embedded (FFPE) samples). The cohorts consisted of 359 consecutive patients and 130 consecutive patients that underwent curative resection for ICC between 2009 and 2013 and between 2014 and 2015, respectively, at the Liver Surgery Department, Zhongshan Hospital, Fudan University. Curative resection was defined as complete resection of tumor nodules, with cancer-free tumor margins shown by histological examination, and resection of regional lymph nodes, including the hilar, hepatoduodenal-ligament and caval lymph nodes, with no cancerous thrombus in the portal vein (main trunk or major branches), hepatic veins or bile duct.^17^ Patients with further lymph node involvement were considered to have distant metastasis and were excluded from the study.^18^ Tumor differentiation was graded histologically according to the Edmondson–Steiner criteria.^19^ Liver function was graded according to the Child-Pugh system. Tumor stage was determined according to the 2017 International Union against Cancer tumor, node, metastasis system.

The clinicopathological characteristics of the patients are listed in online supplemental table 1. The present study includes follow-up data collected through December 2018. The follow-up procedures are described in detail elsewhere.^20 21^

10.1136/jitc-2020-001946.supp1 Supplementary data

### Cell lines and animals

We used three human ICC cell lines: HuCCT1, SG231 (kindly provided by Dr Robert Anders at Johns Hopkins University) and RBE (purchased from the Chinese Academy of Sciences Shanghai Branch Cell Bank, Shanghai, China). These cell lines were routinely maintained in a humidified incubator at 37°C, 5% CO_2_ in RPMI 1640 supplemented with 10% heat-inactivated fetal bovine serum (FBS). We obtained male NOD-*Prkdc^scid^ IL2rg^tm1^*/Bcgen mice aged 4–6 weeks from Beijing Biocytogen and maintained them under specific pathogen-free conditions. Humane care was provided for all animals in accordance with the criteria described in the Guide for the Care and Use of Laboratory Animals (National Institutes of Health publication 86–23, revised 1985).

### TAN and TAM isolation

For TANs isolation, fresh ICC tissues were sliced into small pieces and digested in RPMI 1640 supplemented with 0.05% collagenase IV (Sigma-Aldrich), 0.002% DNase I (Roche) and 20% FBS at 37°C for 30 min. We filtered dissociated cells through a 150 µm mesh and then these cells were centrifuged at 2500 rpm for 20 min with 1 mL cell suspension and 10 mL Ficoll-Hypaque in a 15 mL tube. Thereafter, the leukocytes were harvested and CD66b+ (human neutrophils) were isolated using the EasySep PE Selection Kit (STEMCELL Technologies, Vancouver, Canada) according to the manufacturer’s protocol.

For TAMs isolation, tumor tissues were harvested and digested and single-cell suspensions were collected as mentioned before.^22^ The leukocytes were harvested and CD14+ macrophages were isolated using CD14 MicroBeads (Miltenyi Biotec) according to the manufacturer’s protocol (online supplemental figure 2).

10.1136/jitc-2020-001946.supp2 Supplementary data

### Co-cultured TANs and TAMs

TANs and TAMs derived from the same patient were seeded to achieve a combined density of 1×10^6^ (TAN:TAM=1:1) in a 15 cm tissue culture plate. In parallel, as controls, TANs and TAMs were cultured individually at a density of 1×10^6^. After incubation for 12 hours, co-cultured cells and controls were trypsinized and single-cell suspensions were separated by flow cytometry. Supernatants were collected as conditioned media (CM) from these cultured cells after incubation for 12 hours.

### Opal multiplex immunofluorescent staining

Tumor tissue sections were blocked with 3% hydrogen peroxide in Tris Buffered Saline with Tween 20 (TBST) for 5 min, and then incubated with the primary rabbit antibody for CD66b (BD Biosciences, clone G10F5; 1:500) for 30 min. Slides were then incubated using the antimouse+rabbit HRP-polymer detection system (Akoya Biosciences) for 5 min each step, before visualization using Opal520 TSA (1:50) for another 5 min. Following this, antigen retrieval was conducted again to prepare the slides for the next antibody. Using this Opal staining method, all samples were stained sequentially with the primary mouse antibody for CD68 (Abcam, clone KP1; 1:1000) visualized with Opal620 TSA (1:50), primary rabbit antibody for oncostatin M (OSM) (Abcam, 1:100) visualized with Opal690 TSA (1:50), primary rabbit antibody for interleukin (IL)-11 (Abcam, clone EPR5446; 1:500) visualized with Opal650 TSA (1:50). Slides were counterstained with 4',6-Diamidino-2-Phenylindole Dihydrochloride (DAPI) (1:2000) for nuclei visualization, and subsequently coverslipped using the VectaShield Hardset mounting media.

### Multispectral image acquisition and analysis

Tumor tissue sections that underwent multiplex fluorescent staining for each fluorophore were imaged using the Vectra Polaris imaging system (Akoya Biosciences) under the appropriate fluorescent filters (green for Opal 520, red for Opal 620 and DAPI) in order to produce the spectral library required for multispectral analysis. A whole slide scan of the multiplex tissue sections produced multispectral fluorescent images visualized in Phenochart (Akoya Biosciences) and imaging at 20× power for further analysis. Analysis of the multispectral images was conducted using inForm image analysis (Akoya Biosciences). Representative images of each sample used to establish tissue segmentation and cell segmentation algorithms were applied to batch analysis of all high power multispectral images. The software was then trained to segment the tissue categories into the cell components: the nuclei, cytoplasm and membrane of each cell. The positivity threshold of each marker was then determined and recorded for further data analysis. Once the algorithm was completed, all images were imported into inForm, and run as a batch.

### Quantification of the spatial distribution of TANs and TAMs

We represented the relative spatial distributions of TANs and TAMs in each specimen with a bivariate point pattern characterized by the bivariate G(r) and K(r) functions. The bivariate G(r) function is a probability function of the nearest neighbor distances within a given radius and is defined as follows:

G(r)=sum[*i*,*j*] I(d[*i*,*j*]≤r) e[*i*,*j*]/n

The bivariate K(r) function is the expected number of cells appearing within the radius and is defined as follows:

K(r)=(α/(n×(n−1)))×sum[*i*,*j*] I(d[*i*,*j*]≤r) e[*i*,*j*]

In both functions, d[*i*,*j*] is the distance between two points, I(d[*i*,*j*]≤r) is the logical decision function within the radius, α is the acreage, n is the number of cells and r is the radius of the area in which the function is evaluated. For the null hypothesis, we assumed that the different immune cell subpopulations are independent of each other with no tendency to aggregate and that the distances between nearest neighbors from different subpopulations follow a Poisson distribution. We used the toolbox ‘spatstat’ in R to calculate the G(r) and K(r) functions.

### Statistical analysis

Data are presented as the mean±SD of three or more independent experiments. Quantitative data were compared using Student’s t-tests. Categorical data were compared using χ^2^ tests or Fisher’s exact tests. Correlation analyses were used to analyze the relationships among the expression levels of CD66b, CD68 and p-STAT3. Overall survival (OS) and cumulative recurrence rates were analyzed by Kaplan-Meier analysis and log-rank tests. Univariate and multivariate analyses were performed using Cox proportional hazards regression models. P values <0.05 were considered statistically significant. All statistical analyses were performed using SPSS V.16.0 for Windows.

### Other materials and methods

Details of the vector construction and cell transfections; RNA isolation; quantitative reverse transcription (qRT)-PCR and RT^2^ profiler PCR array experiments; western blot analyses; ELISA; TMA and immunohistochemistry experiments; evaluation of immunohistochemical variables; cell-proliferation, Matrigel-invasion and colony-formation assays and in vivo assays of tumor growth and metastasis are described in the online supplemental materials and methods.

10.1136/jitc-2020-001946.supp3 Supplementary data

## Results

### TANs and TAMs were co-distributed within ICC tumors

**Figure 1:**
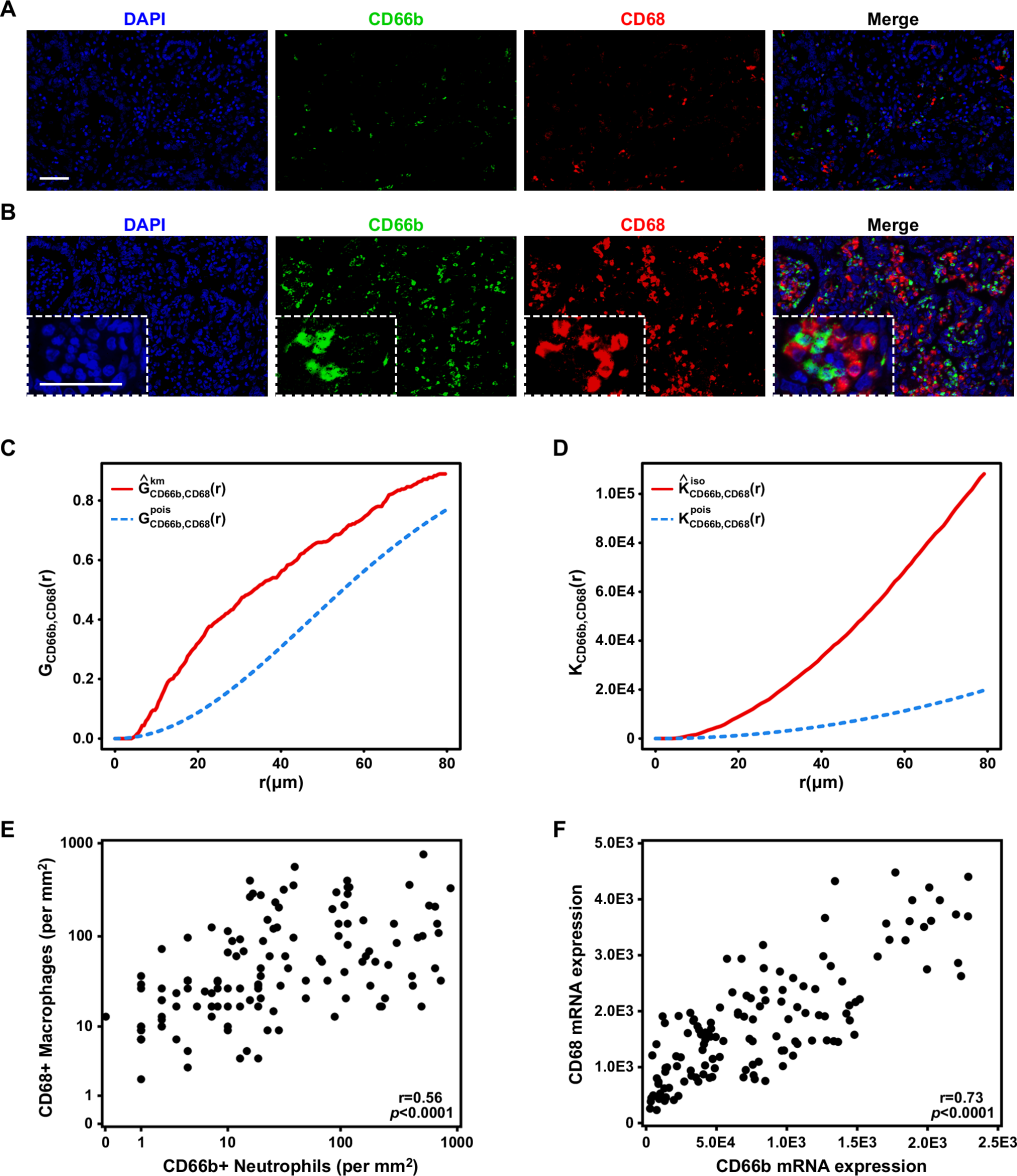
Correlation of tumor-associated neutrophils (TANs) and tumor-associated macrophages (TAMs) in intrahepatic cholangiocarcinoma (ICC). Representative immunofluorescence images of CD66b (green), CD68 (red) and DAPI (blue) staining showing (A) a small cluster and (B) a larger cluster of TANs and TAMs in ICC. Scale bars: 50 μm. (C) G(r) and (D) K(r) functions showed the theoretical distribution (blue line) and the actual distribution (red line) of distances between TANs and TAMs. (E) A scatter plot illustrated the positive correlation between the numbers of TANs and TAMs. (F) A scatter plot illustrated the positive correlation between the RNA expression levels of CD66b and CD68.

First, we performed multiplex immunofluorescent staining for CD66b+ TANs and CD68+ TAMs in whole tumor sections from 130 patients with ICC. We observed that the CD66b+ TANs were in almost all cases located in close proximity to the CD68+ TAMs, forming small clusters in about two-thirds of the samples (figure 1A) and larger clusters in the remaining one-third of the samples (figure 1B). Combined G(r) and K(r) functions supported the observation that TANs and TAMs aggregated closer together than would be expected based on independent spatial distributions (figure 1C, D). Quantification of CD66b and CD68 in the tumor tissues demonstrated that the densities of the CD66b+ TANs were correlated with those of the CD68+ TAMs (figure 1E). To confirm those observations, we performed qRT-PCR using RNA samples from the same patients and found that the levels of CD66b and CD68 messenger RNAs (mRNAs) were also correlated (figure 1F). We also confirmed that these TAN-TAM clusters were not specific to CD45+ immune cells trapped in the stroma (online supplemental figure 1).

### Interaction between TANs and TAMs promoted ICC cell proliferation, invasion and colony formation in vitro and tumor growth and metastasis in vivo

**Figure 2:**
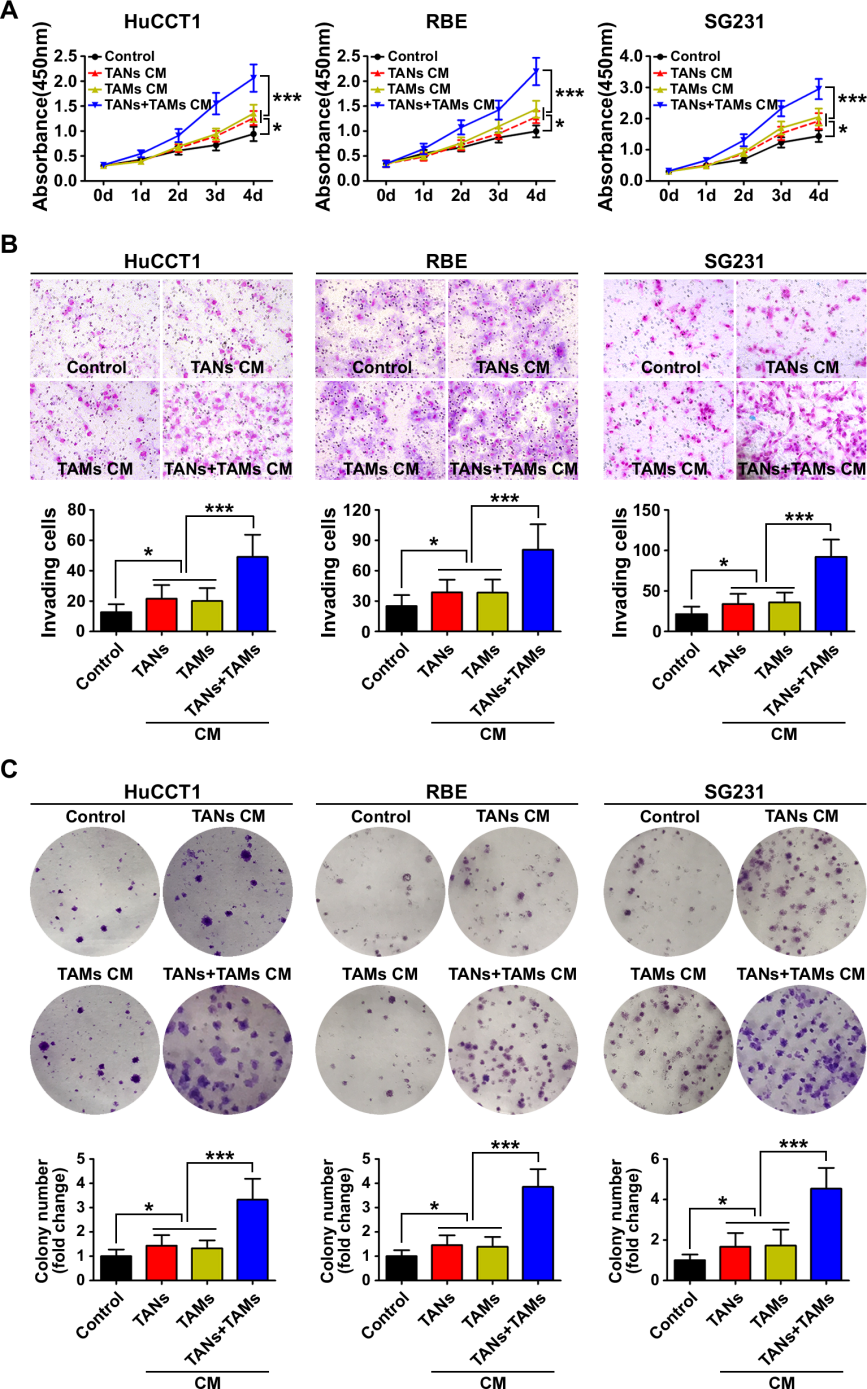
Biological effect of the interaction between tumor-associated neutrophils (TANs) and tumor-associated macrophages (TAMs) on intrahepatic cholangiocarcinoma (ICC) cells. (A) CCK8 cell proliferation assay showed that conditioned medium (CM) from TAN and TAM promoted ICC cell proliferation. Data are shown as the mean±SD (n=6 for each group). (B) Invasion of ICC cells cultured with CM from TAN and TAM compared with that of control ICC cells. The graphs show the number of invasive cells after 48 hours. Data are shown as the mean±SD (n=6 for each group). (C) Cultured with CM from TAN and TAM increased the colony-formation activity of ICC cells Data are shown as the mean±SD (n=6 for each group). *P<0.05, ***p<0.001.

We used cell-proliferation, Matrigel-invasion and colony-formation assays to determine the impacts of TANs and TAMs on ICC cells in vitro. We found that the proliferation, invasion and colony-formation abilities of HuCCT1 cells were enhanced when the cells were cultured in CM from TAN or TAM monocultures (p<0.05). The enhancement of proliferation, invasion and colony formation was increased further when the HuCCT1 cells were cultured in CM from co-cultures of TANs and TAMs (p<0.001; figure 2A–C). Similar results were obtained with RBE cells and SG231 cells (figure 2A–C).

**Figure 3:**
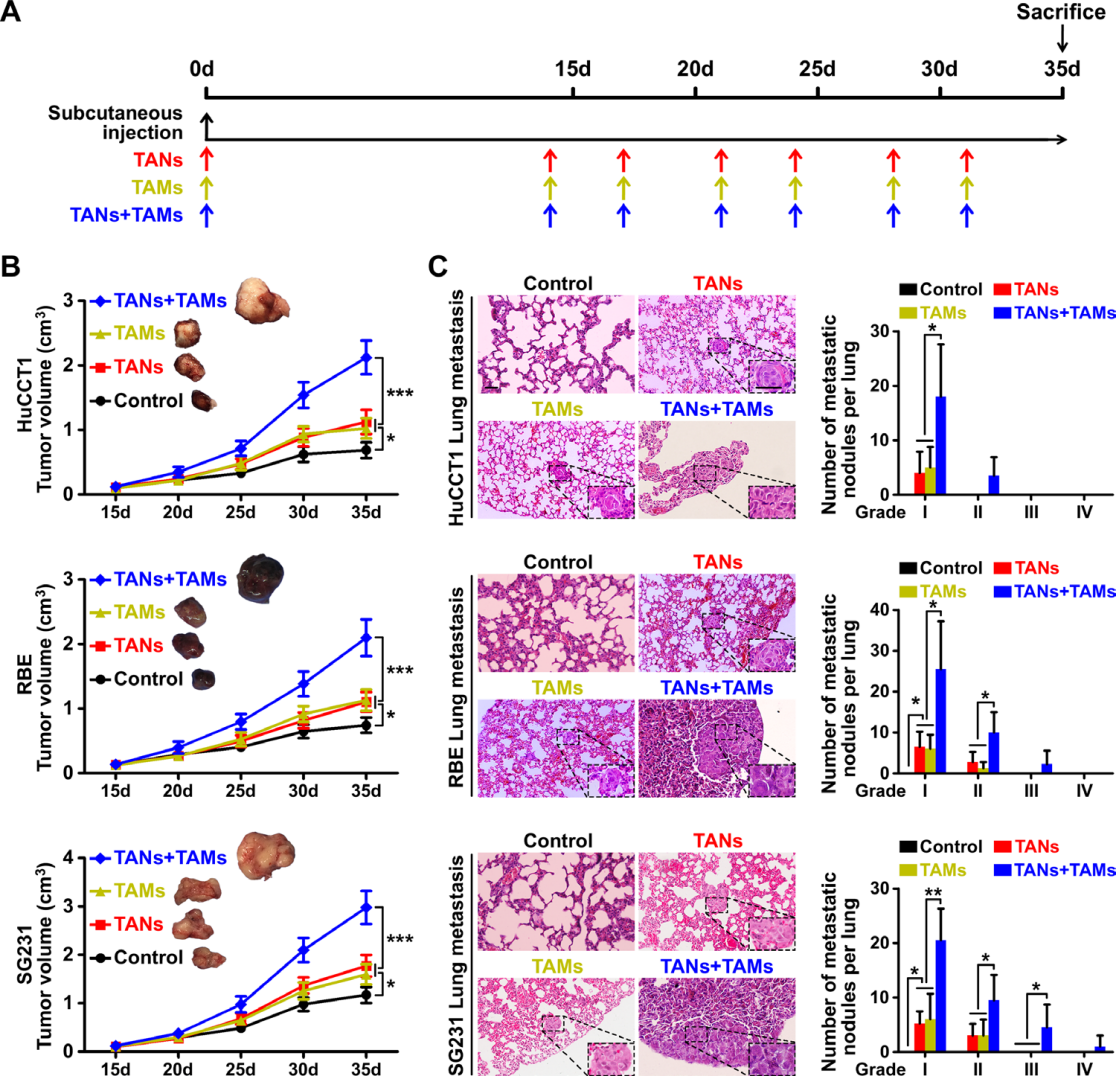
Influence of the interaction between tumor-associated neutrophils (TANs) and tumor-associated macrophages (TAMs) on intrahepatic cholangiocarcinoma (ICC) growth and metastasis. (A) Schematic of the mouse xenograft experiments. Control group: 1×10^7^ ICC cells were injected into the subcutaneous space of the upper left flank region of NOD-*Prkdc^scid^ IL2rg^tm1^*/Bcgen mice at day 0. TANs group: 1×10^7^ ICC cells were co-injected with 1×10^6^ TANs into the subcutaneous space of the upper left flank region of mice at day 0, and TANs were injected into the tumor at the indicated time (red arrows). TAMs group: 1×10^7^ ICC cells were co-injected with 1×10^6^ TAMs into the subcutaneous space of the upper left flank region of mice at day 0, and TAMs were injected into the tumor at the indicated time (yellow arrows). TANs+TAMs group: 1×10^7^ ICC cells were co-injected with 1×10^6^ TANs and TAMs mixture (TANs:TAMs=1:1) into the subcutaneous space of the upper left flank region of mice at day 0, and TANs and TAMs mixture were injected into the tumor at the indicated time (blue arrows). All mice were monitored once every 5 days and killed 5 weeks later. (B) Xenografts containing TANs and TAMs produced greater tumor volume and more pulmonary metastasis (C) than xenografts composed of ICC cells alone, *p<0.05, **p<0.01, ***p<0.001. Scale bars: 50 μm. Data are shown as the mean±SD (n=4 for each experiment mice group).

We designed a mouse xenograft model to investigate the influence of TANs and TAMs on ICC growth and metastasis in vivo (figure 3A, online supplemental figure 3). We found that xenografts consisting of ICC cells and either TANs or TAMs produced larger tumor volumes than xenografts consisting of ICC cells alone (figure 3B, online supplemental figure 4). The inclusion of TANs or TAMs in the xenografts also resulted in higher rates of pulmonary metastasis compared with the rate in the absence of exogenous TANs or TAMs (figure 3C). When TANs and TAMs were both included in the xenografts, the tumor volumes and rate of pulmonary metastasis were even higher than those of the xenografts containing either TANs or TAMs alone (figure 3B, C).

### TANs and TAMs produced OSM and IL-11, respectively, in co-culture

**Figure 4:**
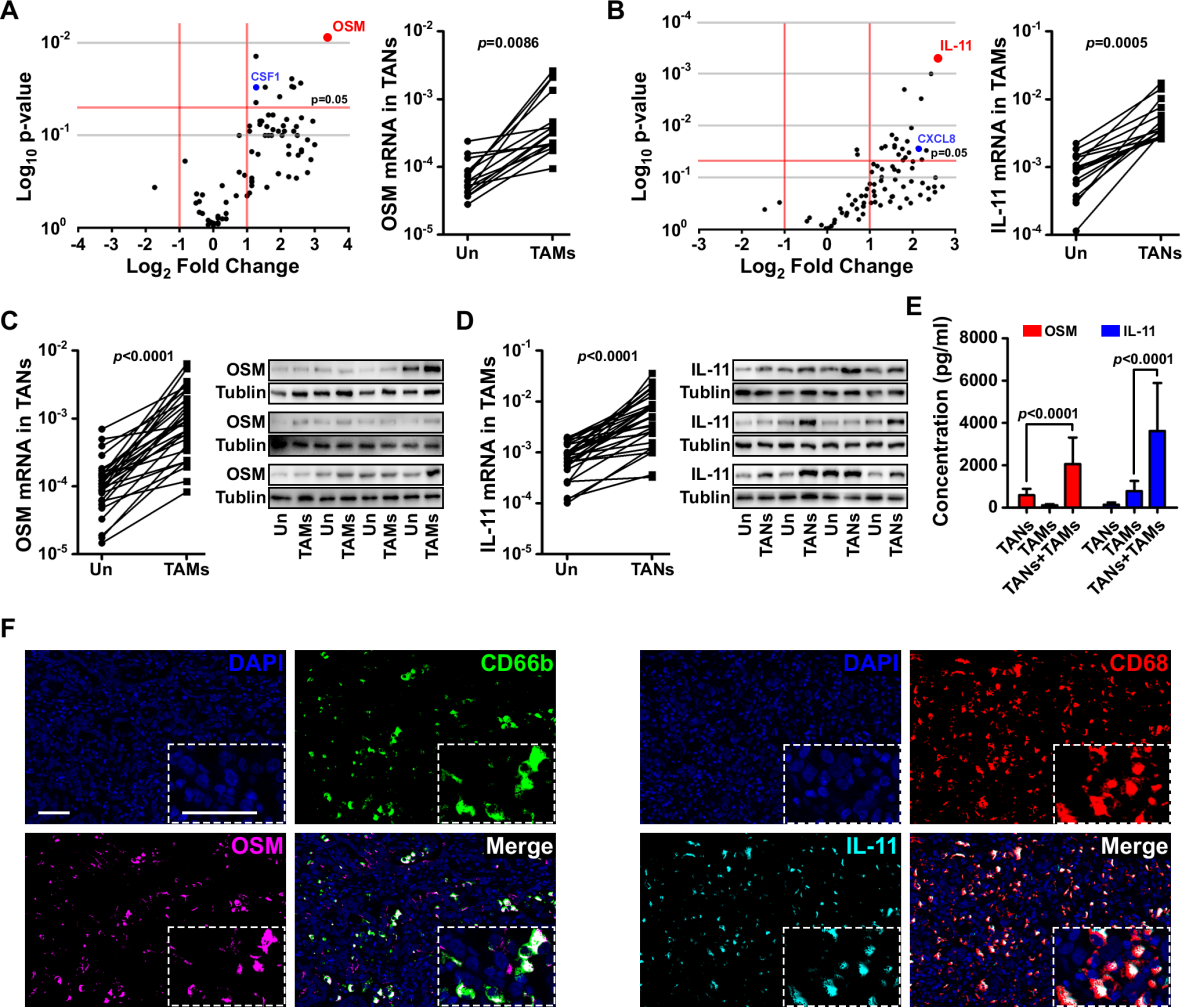
Tumor-associated neutrophils (TANs) and tumor-associated macrophages (TAMs) produced oncostatin M (OSM) and interleukin (IL)-11, respectively, in co-culture. (A) A PCR array showed altered expression of cytokines and chemokines by TANs after co-culture with TAMs. (B) A PCR array showed altered expression of cytokines and chemokines by TAMs after co-culture with TANs. (C) quantitative reverse transcription (qRT)-PCR and western blot analysis validated the messenger RNA (mRNA) and protein levels of OSM in TANs after co-culture with TAMs. (D) qRT-PCR and western blot analysis validated the mRNA and protein levels of IL-11 in TAMs after co-culture with TANs. (E) ELISA confirmed the presence of OSM and IL-11 in conditioned media obtained from co-cultured TANs and TAMs. (F) Representative images of OSM expression (magenta) in TANs (CD66b, green cells), and IL-11 expression (cyan) in macrophages (CD68, red cells) obtained by opal multiplex immunofluorescent staining of intrahepatic cholangiocarcinoma (ICC) samples. Scale bars: 50 μm.

We used a PCR array to quantify the expression of a panel of cytokines and chemokines in co-cultures of TANs and TAMs after 12 hours. We used separate cultures of TANs and TAMs as controls. Of all the cytokines and chemokines evaluated, OSM and IL-11 were the ones most abundantly expressed by TANs and TAMs, respectively, in co-culture (figure 4A, B).

To confirm the expression of OSM and IL-11 in ICC-associated TANs and TAMs, we examined the mRNA and protein levels of OSM and IL-11 in TANs and TAMs isolated from an additional 30 patients with ICC. We found that the level of OSM expression by patient-derived TANs was higher when those cells were co-cultured with patient-derived TAMs than when the TANs were cultured alone (figure 4C). Likewise, the level of IL-11 expression in the patient-derived TAMs was higher in co-cultures with patient-derived TANs than in TAM monocultures (figure 4D, online supplemental figure 5). ELISA results confirmed that the levels of both cytokines in CM from co-cultures of patient-derived TANs and TAMs were higher than those in CM from monocultures of TANs or TAMs (figure 4E). Furthermore, opal multiplex immunofluorescent staining confirmed that OSM was preferentially expressed by TANs, whereas IL-11 was preferentially expressed by TAMs, in the ICC samples in which those cells formed larger clusters (figure 4F).

### OSM and IL-11 activated STAT3 signaling and promoted proliferation, invasion and colony formation in ICC cells

**Figure 5:**
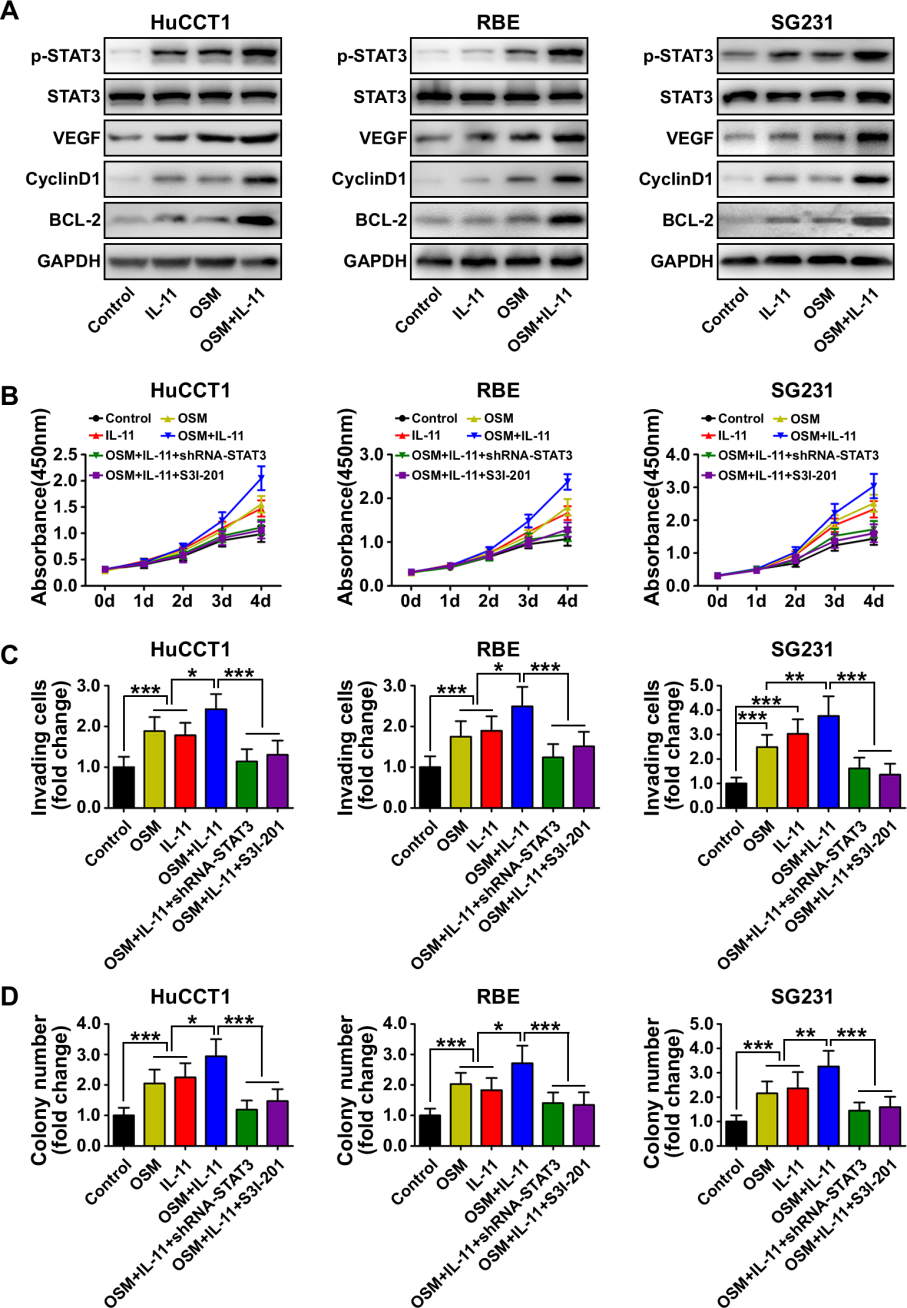
Biological effect and mechanism of oncostatin M (OSM) and interleukin (IL)-11 in intrahepatic cholangiocarcinoma (ICC) cells. (A) Western blot analysis showed the phosphorylation state of STAT3 and downstream STAT3 targets including vascular endothelial growth factor (VEGF), cyclin D1 and BCL-2 in ICC cells after OSM and IL-11 treatment. (B) CCK8 cell proliferation assay showed ICC cell proliferation in the indicated treatment groups. Data are shown as the mean±SD (n=6 for each group). (C) Matrigel-invasion assay showed the invasion of ICC cells in the indicated treatment groups. Data are shown as the mean±SD (n=6 for each group). (D) Colony-formation activity of ICC cells in the different treatment groups. Data are shown as the mean±SD (n=6 for each group). *P<0.05, **p<0.01, ***p<0.001.

In order to investigate the roles of OSM and IL-11 in ICC cells, we analyzed the phosphorylation of Akt, ERK1/2, p38 MAPK, JNK, p65 (nuclear factor kappa B pathway) and STAT3 in ICC cells treated with or without OSM (5 ng/mL) and IL-11 (10 ng/mL) to determine the kinase profiles of distinct signaling pathways. We found that p-STAT3^Tyr705^ was upregulated in response to OSM or IL-11 treatment in all three ICC cell lines and was further upregulated in response to combined OSM and IL-11 treatment (figure 5A). The expression of downstream STAT3 targets including vascular endothelial growth factor, cyclin D1 and BCL2 apoptosis regulator (BCL-2) was also increased in response to OSM and IL-11 treatment (figure 5A). Separate or combined OSM and IL-11 treatments did not significantly affect Akt, ERK1/2, p38 MAPK, JNK or p65 phosphorylation in the ICC cell lines (online supplemental figure 6).

We next tested the effects of OSM and IL-11 on ICC cellular behaviors. Cell functional assays showed that OSM or IL-11 treatment induced proliferation, invasion and colony formation in ICC cells (figure 5B–D). Treatment with both cytokines simultaneously further enhanced each behavior, whereas STAT3 knockdown in the ICC cells or pretreatment of the ICC cells with the STAT3 inhibitor S3I-201 suppressed all three behaviors (figure 5B–D). Those results suggested that OSM and IL-11 promote ICC cell proliferation, invasion and colony formation by activating STAT3 signaling.

### Inhibition of STAT3 signaling abolished the protumor effect of TANs and TAMs interaction on ICC

**Figure 6:**
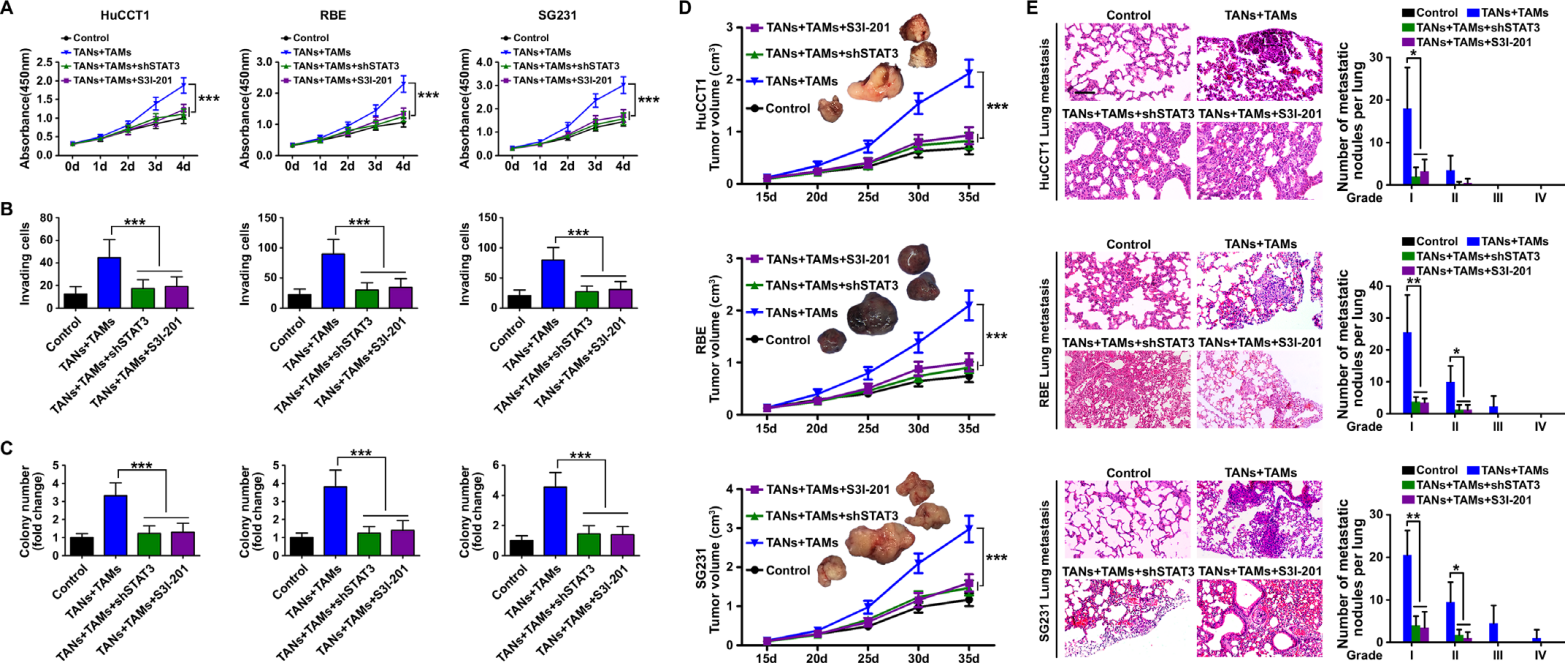
The role of STAT3 signaling in the protumor effect of the interaction between tumor-associated neutrophils (TANs) and tumor-associated macrophages (TAMs) in intrahepatic cholangiocarcinoma (ICC). (A) CCK8 cell proliferation assay showed ICC cell proliferation after treatment with TANs and TAMs, shRNA-STAT3 or the STAT3 inhibitor S3I-201 (200 μM). Data are shown as the mean±SD (n=6 for each group). (B) Matrigel-invasion assay showed ICC cell invasion after treatment with TANs and TAMs, shRNA-STAT3 or the STAT3 inhibitor S3I-201. Data are shown as the mean±SD (n=6 for each group). (C) Colony-formation activity of ICC cells after treatment with TANs and TAMs, shRNA-STAT3 or the STAT3 inhibitor S3I-201, ***p<0.001. Data are shown as the mean±SD (n=6 for each group). (D) Tumor volume and (E) pulmonary metastasis of mouse xenografts composed of ICC cells treated with TANs and TAMs with or without shRNA-STAT3 or the STAT3 inhibitor S3I-201. *P<0.05, **p<0.01, ***p<0.001. Scale bars: 100 μm. Data are shown as the mean±SD (n=4 for each experiment mice group).

We next asked whether the effect of TANs and TAMs on ICC requires STAT3 signaling. We found that STAT3 knockdown in ICC cells or pretreatment of ICC cells with S3I-201 alleviated the protumor effects of TANs and TAMs on those cells in vitro (figure 6A–C) and promoted tumor growth and metastasis in mouse xenografts (figure 6D, E, online supplemental figure 7). Those results suggested that the effects of TANs and TAMs interaction on ICC tumor progression rely on STAT3 signaling.

### TAN, TAM and p-STAT3 levels were correlated in ICC tissues and predictive of prognosis

**Figure 7:**
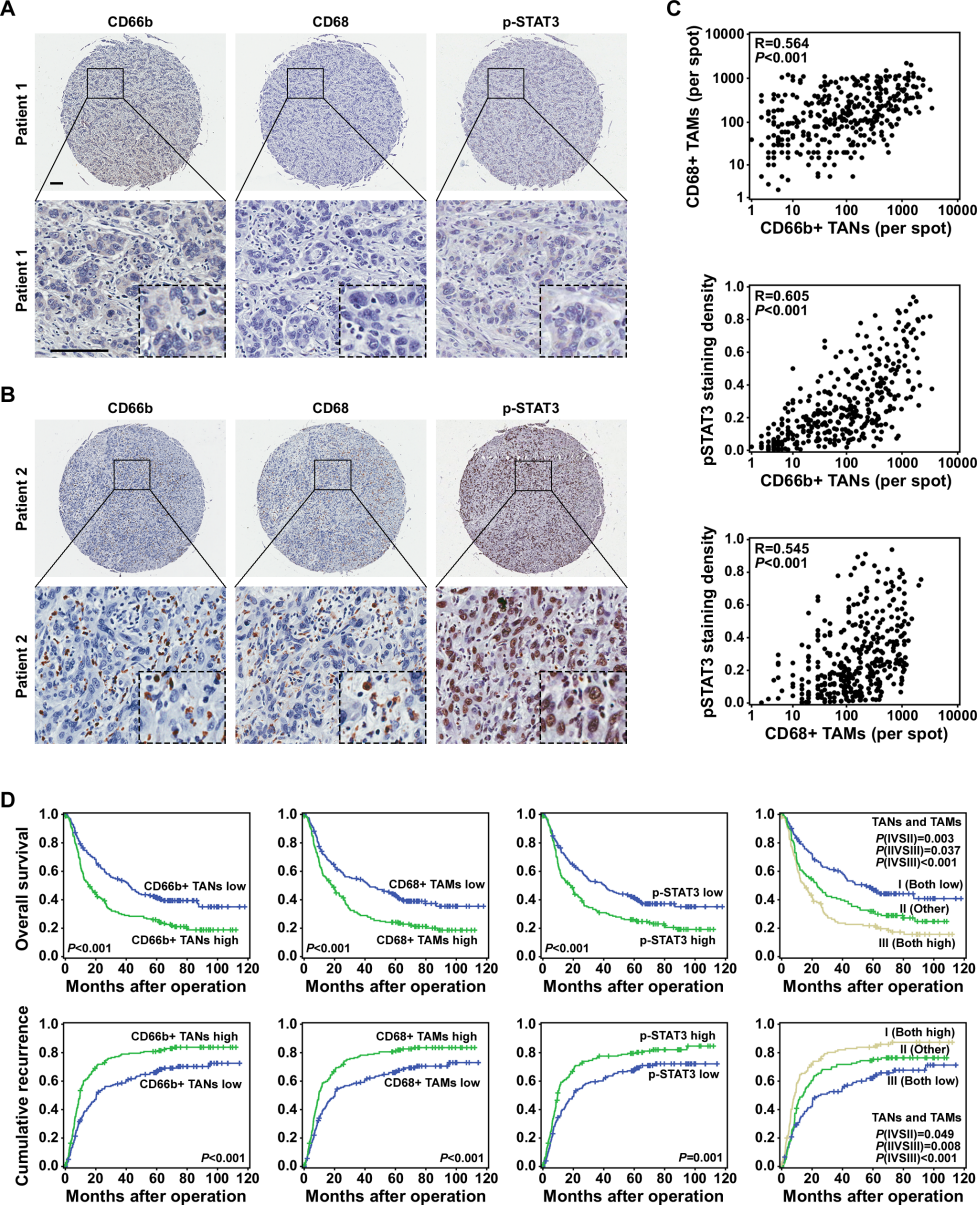
Tumor-associated neutrophil (TAN) levels were correlated with tumor-associated macrophage (TAM) infiltration, p-STAT3 expression and prognosis in patients with intrahepatic cholangiocarcinoma (ICC). (A and B) Expression of CD66b, CD68 and p-STAT3 in representative ICC tumor samples, Scale bars: 100 μm. (C) A scatter plot illustrated the correlation between the numbers of TANs and TAMs and p-STAT3 expression. (D) The numbers of TANs and TAMs, p-STAT3 expression and the combination of those factors (TANs/TAMs) was predictive of prognosis in patients with ICC.

We measured the numbers of TANs and TAMs and the level of p-STAT3 expression using a TMA composed of primary tumor tissues from 359 patients with ICC. Representative cases are shown in figure 7A, B. Tumors with greater TAN and TAM infiltration tended to have higher levels of p-STAT3 expression, and vice versa (figure 7C). Their densities were all associated with disease progression (online supplemental figure 8).

Kaplan-Meier survival analysis showed that patients with greater TAN and/or TAM infiltration had lower OS rates and higher cumulative recurrence rates than those with less TAN and/or TAM infiltration (figure 7D). Furthermore, the expression of p-STAT3 was correlated with OS (p<0.001, HR=1.59) and time to recurrence (p<0.001, HR=1.5; table 1). Multivariate analyses revealed that in addition to gamma-glutamyl transferase levels, tumor size, tumor number and lymphatic metastasis, the number of infiltrated TANs and TAMs, the coindex (TANs/TAMs) and the p-STAT3 expression level were independent prognostic factors for both OS and time to recurrence (table 1).

**Table 1:** Univariate and multivariate analyses of prognostic factors in ICC (n=359)

Variable	TTR		OS	
	HR (95% CI)	P value	HR (95% CI)	P value
**Univariate analysis**				
Age, year (≤50 vs >50)	1.06 (0.79 to 1.43)	0.689	1.29 (0.94 to 1.78)	0.116
Sex (female vs male)	1.17 (0.92 to 1.50)	0.2	1.07 (0.83 to 1.38)	0.583
HBsAg (negative vs positive)	1.02 (0.79 to 1.33)	0.872	0.72 (0.54 to 0.96)	0.025
AFP, ng/mL (≤20 vs >20)	0.99 (0.68 to 1.43)	0.941	0.84 (0.56 to 1.26)	0.396
CA19-9 (≤36 vs >36)	1.51 (1.18 to 1.94)	0.001	1.90 (1.46 to 2.48)	0
GGT, U/L (≤54 vs >54)	1.63 (1.28 to 2.09)	0	1.92 (1.48 to 2.48)	0
Liver cirrhosis (no vs yes)	1.02 (0.77 to 1.36)	0.88	0.83 (0.61 to 1.12)	0.226
Tumor size, cm (≤5 vs >5)	1.59 (1.25 to 2.04)	0	1.65 (1.28 to 2.14)	0
Tumor number (single vs multiple)	2.36 (1.82 to 3.05)	0	2.66 (2.03 to 3.47)	0
Microvascular/Bile duct invasion (no vs yes)	1.25 (0.93 to 1.69)	0.139	1.48 (1.10 to 1.97)	0.009
Lymphatic metastasis (no vs yes)	2.41 (1.77 to 3.28)	0	2.70 (1.96 to 3.72)	0
Tumor encapsulation (complete vs none)	1.46 (1.03 to 2.05)	0.033	1.46 (1.01 to 2.11)	0.042
Tumor differentiation (I+II vs III+IV)	1.31 (1.03 to 1.67)	0.028	1.43 (1.11 to 1.84)	0.005
TNM stage (I vs II+III+IV)	2.29 (1.79 to 2.93)	0	2.78 (2.14 to 3.61)	0
CD66b+ TANs (low vs high)	1.61 (1.27 to 2.06)	0	1.72 (1.34 to 2.22)	0
CD68+ TAMs (low vs high)	1.55 (1.21 to 1.97)	0	1.68 (1.30 to 2.16)	0
p-STAT3 (low vs high)	1.50 (1.18 to 1.91)	0.001	1.59 (1.23 to 2.05)	0
TANs and TAMs (both low vs both high)	1.97 (1.46 to 2.67)	0	2.25 (1.64 to 3.08)	0
**Multivariate analysis**				
HBsAg (negative vs positive)	NA	NA	0.29 (0.53 to 0.97)	0.718
CA19-9 (≤36 vs >36)	1.10 (0.85 to 1.43)	0.481	1.39 (1.06 to 1.83)	0.018
GGT, U/L (≤54 vs >54)	1.45 (1.12 to 1.87)	0.005	1.52 (1.16 to 1.99)	0.003
Tumor size, cm (≤5 vs >5)	1.37 (1.06 to 1.78)	0.016	1.36 (1.03 to 1.79)	0.033
Tumor number (single vs multiple)	1.97 (1.50 to 2.59)	0	2.12 (1.61 to 2.81)	0
Microvascular/Bile duct invasion (no vs yes)	NA	NA	1.26 (0.92 to 1.73)	0.146
Lymphatic metastasis (no vs yes)	1.78 (1.29 to 2.45)	0	1.94 (1.38 to 2.71)	0
Tumor encapsulation (complete vs none)	1.43 (1.01 to 2.03)	0.046	1.41 (0.97 to 2.06)	0.073
Tumor differentiation (I+II vs III+IV)	1.25 (0.97 to 1.60)	0.08	1.31 (1.01 to 1.70)	0.042
CD66b+ TANs (low vs high)	1.59 (1.24 to 2.03)	0	1.71 (1.31 to 2.22)	0
CD68+ TAMs (low vs high)	1.52 (1.19 to 1.94)	0.001	1.53 (1.18 to 1.98)	0.001
p-STAT3 (low vs high)	1.48 (1.16 to 1.89)	0.002	1.59 (1.23 to 2.05)	0
TANs and TAMs (both low vs both high)	2.01 (1.48 to 2.73)	0	2.28 (1.64 to 3.18)	0

Cox proportional hazards regression model.

AFP, alpha-fetoprotein; CA19-9, carbohydrate antigen 19-9; GGT, gamma-glutamyl transferase; HBsAg, hepatitis B surface antigen; ICC, intrahepatic cholangiocarcinoma; NA, not available; OS, overall survival; TAM, tumor-associated macrophage; TAN, tumor-associated neutrophil; TNM, tumor, node, metastasis; TTR, time to recurrence.

## Discussion

ICCs exist within complex microenvironments in which stromal cells and different types of immune cells create and maintain inflammatory conditions that promote tumor growth, invasion, and metastasis.^2 23^ Although various stromal cells and immune cells directly or indirectly influence ICC progression by secreting molecules that exert protumor or antitumor functions, the interaction between TANs and TAMs and its effect on cancer progression have not been elucidated. Our experiments revealed that TANs and TAMs interact to promote ICC progression by a mechanism that depends on OSM/IL-11/STAT3 signaling.

We observed that TANs and TAMs were co-distributed and that their local densities were well correlated in clinical ICC samples. Those observations made us interested in exploring the effect of their interaction on ICC cell characteristics. We found that the interaction between TANs and TAMs enhanced ICC cell proliferation, invasion, and colony formation in vitro and tumor growth and metastasis in vivo. Although TANs or TAMs alone could also achieve those effects, the extent of the protumor effects when TANs and TAMs were both present far exceed the effects when either TANs or TAMs were solely present. In addition, we found that CM from indirect co-cultures of TANs and TAMs can also increase ICC cell proliferation, invasion and colony formation in vitro relative to TAN or TAM monocultures, although the response was weaker than that achieved via direct co-cultures (online supplemental figure 9). Thus, we proposed that direct contact between TANs and TAMs may in part account for their signaling to the tumor cells, in combination soluble mediators that will be under further investigation in the future. Those results suggested that there might be cytokines or chemokines induced by the interaction between TANs and TAMs that have the ability to promote ICC progression.

In order to test that hypothesis, we performed PCR array and ELISA experiments to screen for cytokine expression and found that OSM and IL-11 expression was increased in TANs and TAMs, respectively, after those cells were grown together in co-culture. OSM and IL-11 are both IL-6 family cytokines, which are expressed during inflammation and cancer.^24 25^ OSM is expressed by cancer-associated adipose tissue.^26^ In gliomas and breast cancer, OSM-mediated signaling was shown to be associated with poor prognosis and tumor aggressiveness.^26–28^ IL-11 has been investigated in various types of malignancies including gastric, colorectal, pancreatic, breast and endometrial cancers. Its increased expression in tumors is correlated with disease progression and poor prognosis.^25^ We confirmed that OSM and IL-11 were abundantly secreted by co-cultured TANs and TAMs, respectively, but not by ICC cells (online supplemental figure 10), which revealed the paracrine roles of those two cytokines in cooperatively promoting ICC cell proliferation, invasion and colony formation. In addition, we found that TANs also express a serious of chemokines, including CCL2, CCL5 and CSF1, which may mediate TAM infiltration; while some potential TAN-chemoattractants, such as CXCL8 and CSF3, were expressed by TAMs. Some of these chemokines, such as CSF1 and CXCL8 (figure 4A, B), were further increased after TAN-TAM co-culture, which suggested a possible positive feedback loop between TANs and TAMs and responsible for their spatial association.

We investigated the downstream signaling in ICC cells activated by OSM and IL-11. Western blot analysis showed that STAT3 played a crucial role in the enhanced ICC cell proliferation and invasion induced by OSM and IL-11. Knockdown of STAT3 or treatment with a STAT3 inhibitor almost completely abolished the protumor effects induced by the interaction between TANs and TAMs on ICC in vitro and in vivo. Although TANs and TAMs can also secrete some other cytokines, which may signal through STAT3 on ICC cells. OSM and IL-11 were the ones most upregulated expressed after co-culture of TANs and TAMs. Thus, we proposed that the effect of TANs and TAMs on ICCs was mainly dependent on OSM and IL-11-mediated STAT3 signaling activation. STAT3 signaling is frequently activated in cancer cells through genetic or epigenetic mechanisms and plays a key role in regulating many genes that promote inflammation in the tumor microenvironment.^24^ Our results revealed a microenvironment-mediated mechanism of STAT3 signaling activation in ICC, which suggested that interaction between TANs and TAMs contributes to ICC progression by activating STAT3.

The newly identified role of the TANs/TAMs/p-STAT3 network in controlling ICC progression suggested that this signaling network could be a prognostic indicator, as well as a therapeutic target. We confirmed in our ICC patient cohort that levels of TANs, TAMs and p-STAT3 correlated in ICC tissues and all were independent prognostic factors. However, the associations were not absolute: some patients had few infiltrations of TANs and/or TAMs, while exhibited strong tumor p-STAT3 density; some other patients displayed weak tumor p-STAT3 density, although infiltrated by abundant TANs and/or TAMs. This may be due to the intrinsic activation of STAT3 in tumor cells through genetic or epigenetic mechanisms, which can promote the differentiation of suppressive cells in the tumor microenvironment. In spite of this, our in vitro and in vivo study and clinical observation suggested that p-STAT3 activation status in patients with ICC is a consequence of the density of TANs and TAMs. These results also indicated that the components of this signaling network should be explored as therapeutic targets to treat ICC.

Collectively, our work identifies an inflammation/immune-associated cellular, molecular and clinical network involving TANs, TAMs and STAT3 signaling in ICC cells, which controls the tumor progression and patient outcome. These findings support their potential for exploration as therapeutic targets for ICC.
