# Supplementary material for: Tumor-associated neutrophils and macrophages interaction contributes to intrahepatic cholangiocarcinoma progression by activating STAT3
Source: J Immunother Cancer. 2021 Mar 10;9(3):e001946. doi: 10.1136/jitc-2020-001946 (PMC7949476; doi:10.1136/jitc-2020-001946)
Supplement: Supplementary data [file jitc-2020-001946supp001.pdf]

Supplementary Table 1. Clinicopathologic characteristics of patients with intrahepatic cholangiocarcinoma (n=359)

Characteristics	Number (%)
Age, year (≤50 versus >50)	74/285 (20.6/79.4)
Sex (female versus male)	153/206 (42.6/57.4)
HBsAg (negative versus positive)	252/107 (70.2/29.8)
AFP, ng/ml (≤20 versus>20)	317/42 (88.3/11.7)
CA199(⩽36 versus >36)	149/210 (41.5/58.5)
GGT,U/L (≤54 versus>54)	172/187 (47.9/52.1)
Liver cirrhosis (no versus yes)	273/86 (76.0/24.0)
Tumor size,cm(≤5 versus>5)	159/200 (44.3/55.7)
Tumor number (single versus multiple)	258/101 (71.9/28.1)
Microvascular/bile duct invasion (no versus yes)	282/77 (78.6/21.4)
Lymphatic metastasis (no versus yes)	306/53 (85.2/14.8)
Tumor encapsulation (complete versus none)	62/297 (17.3/82.7)
Tumor differentiation (I+II versus III+IV)	181/178 (50.4/49.6)
TNM stage (I versus II+III+IV)	182/177 (50.7/49.3)

Abreviations: AFP, alpha-fetoprotein; GGT, gamma glutamyl transferase; CA 19-9, carbohydrate antigen 19-9; TNM, tumor-node-metastasis.
