# Supplementary material for: Tumor-associated neutrophils and macrophages interaction contributes to intrahepatic cholangiocarcinoma progression by activating STAT3
Source: J Immunother Cancer. 2021 Mar 10;9(3):e001946. doi: 10.1136/jitc-2020-001946 (PMC7949476; doi:10.1136/jitc-2020-001946)
Supplement: Supplementary data [file jitc-2020-001946supp002.pdf]

Supplementary Figures and Legends

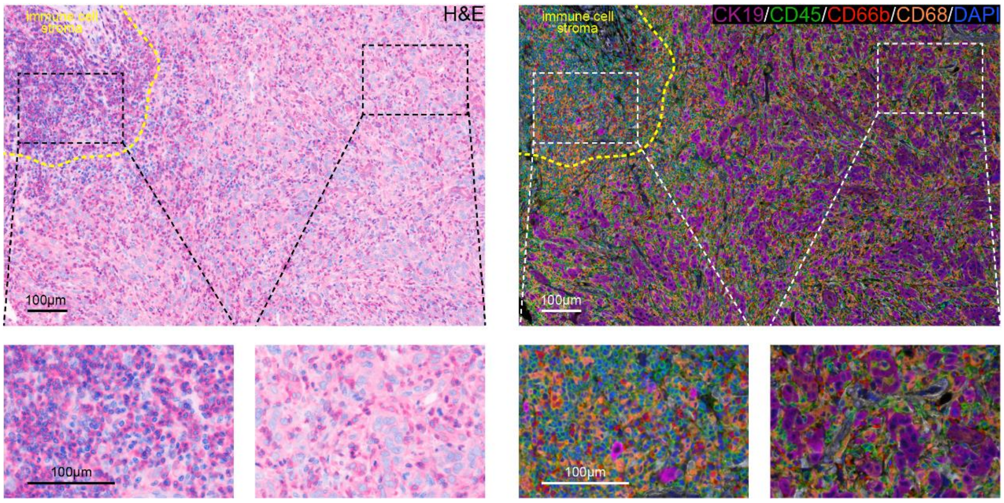

Supplementary Figure 1 H&E and immunofluorescence images of CK19, CD45, CD66b, CD68, and DAPI staining in ICC tissues. The area circled by yellow dotted line indicate the immune cell stroma.

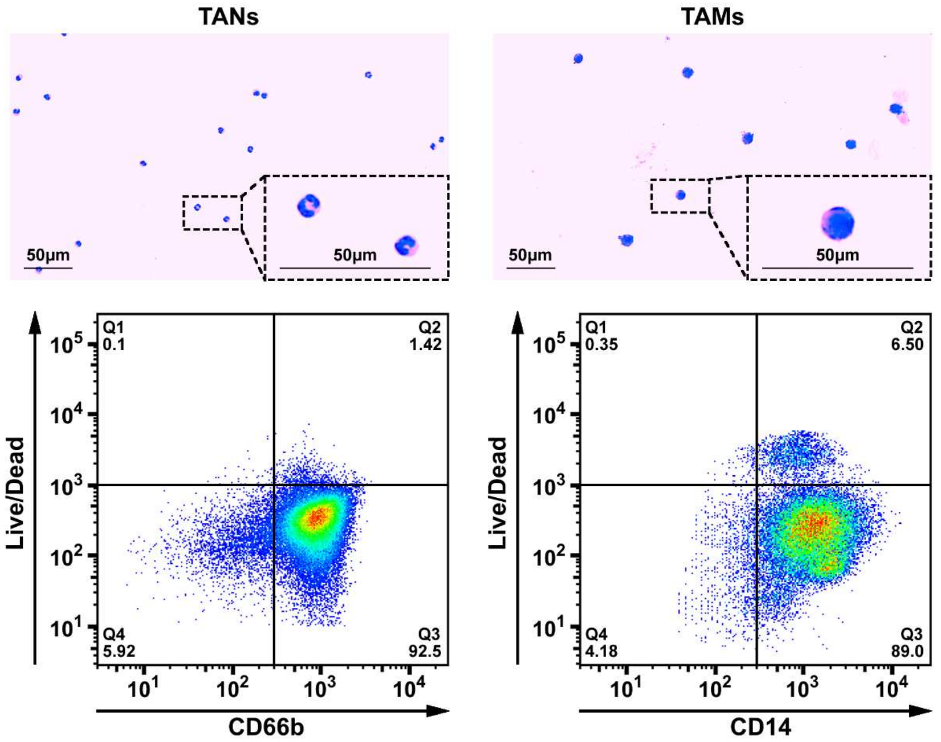

Supplementary Figure 2 Giemsa staining and flow cytometry of isolated TANs and TAMs.

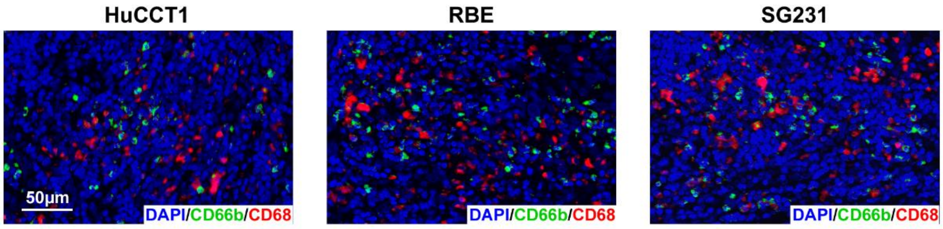

Supplementary Figure 3 Multiplex immunofluorescent staining to detect injected TAN and TAM distribution in the TANs+TAMs group of mouse xenograft model of ICC.

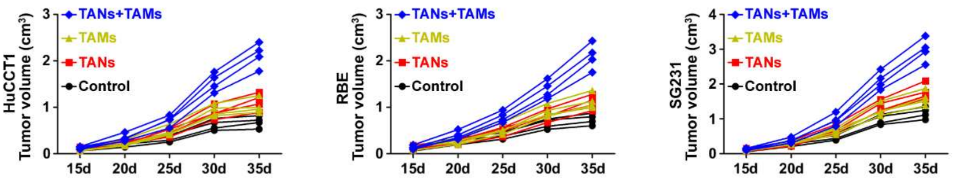

Supplementary Figure 4 Individual curves of tumor volume derived from mouse xenograft model of ICC, related to **Figure 3B**.

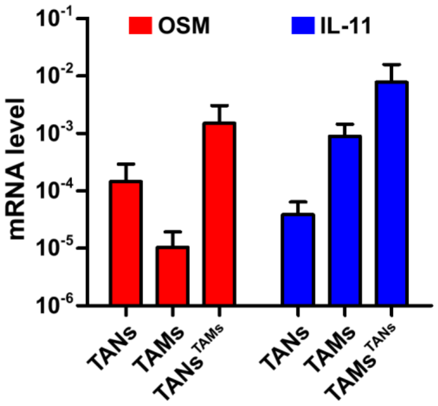

Supplementary Figure 5 mRNA levels of OSM and IL-11 produced when TAMs and TANs were cultured alone and their co-culture.

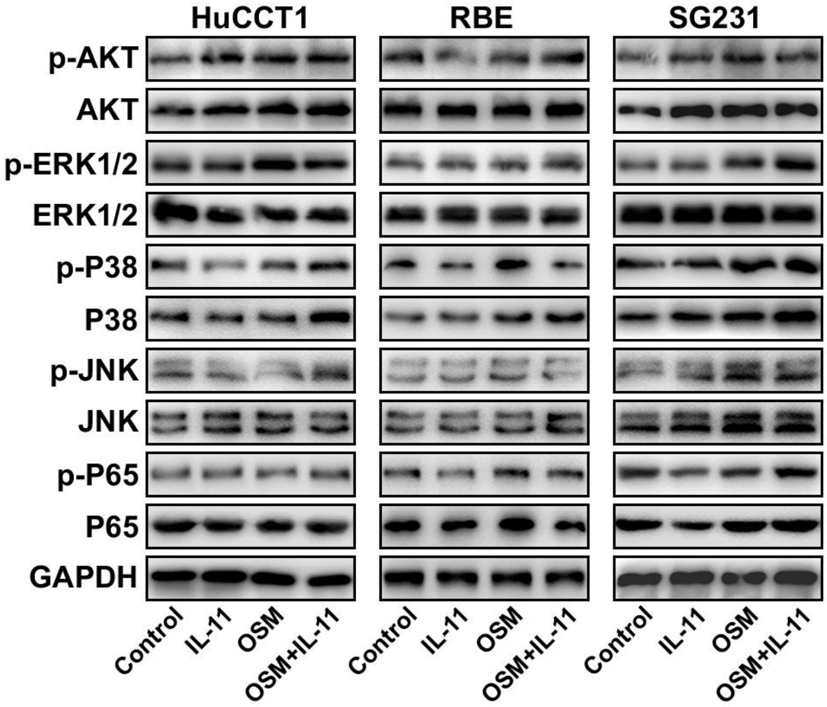

Supplementary Figure 6 Western blot showed Akt, ERK1/2, p38, JNK, and p65 phosphorylation in ICC cells.

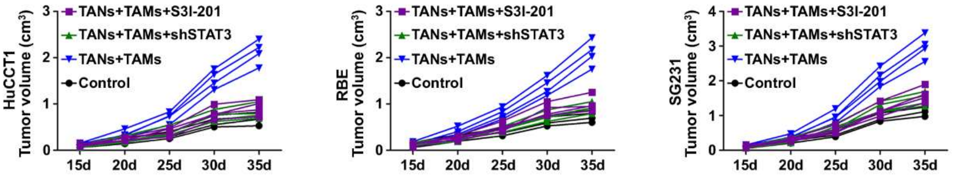

Supplementary Figure 7 Individual curves of tumor volume derived from mouse xenograft model of ICC, related to **Figure 6D**.

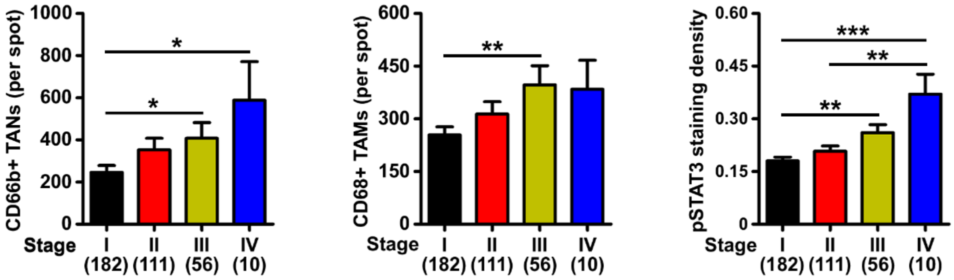

Supplementary Figure 8 The association between TAN and TAM density, and pSTAT3 expression and tumor stage in ICC patients, **P*<0.05, ***P*<0.01, ****P*<0.001.

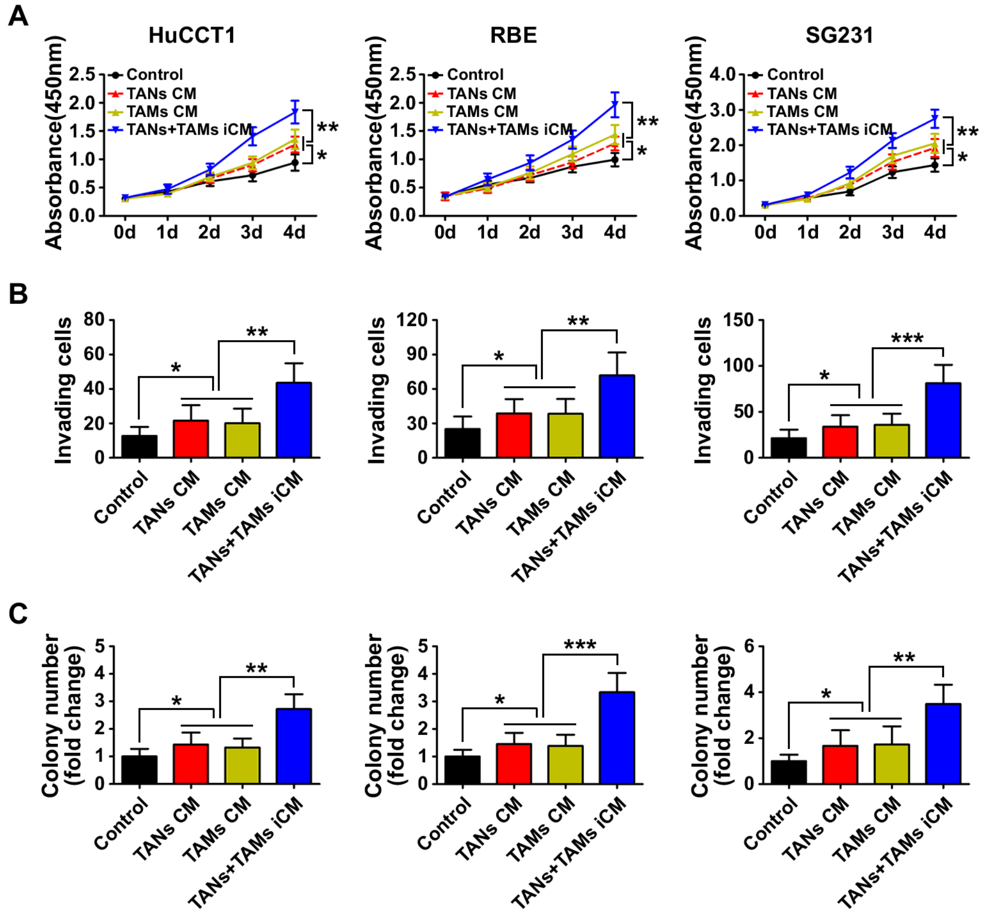

Supplementary Figure 9 Biological effect of the indirect cocultures of TANs and TAMs on ICC cells. (A) CCK8 cell proliferation assay showed that conditioned medium (CM) from TANs, TAMs or indirect cocultures of TANs and TAMs promoted ICC cell proliferation, **P*<0.05, ***P*<0.01. Data are shown as the mean ± SD (n=6 for each group). **(B)** Invasion of ICC cells cultured with CM from TANs, TAMs or indirect cocultures of TANs and TAMs compared with that of control ICC cells. The graphs show the number of invasive cells after 48 h, **P*<0.05, ***P*<0.01, ****P*<0.001. Data are shown as the mean ± SD (n=6 for each group). **(C)** cultured with CM from TANs, TAMs or indirect cocultures of TANs and TAMs increased the colony formation activity of ICC cells, **P*<0.05, ***P*<0.01, ****P*<0.001. Data are shown as the mean ± SD (n=6 for each group).

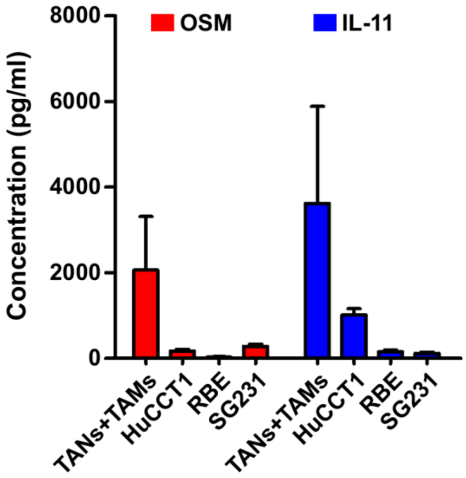

Supplementary Figure 10 Enzyme-linked immunosorbent assay showed the secreted level of OSM and IL-11 in conditioned media obtained from co-cultured TANs and TAMs, and three ICC cells.
