# Supplementary material for: Tumor-associated neutrophils and macrophages interaction contributes to intrahepatic cholangiocarcinoma progression by activating STAT3
Source: J Immunother Cancer. 2021 Mar 10;9(3):e001946. doi: 10.1136/jitc-2020-001946 (PMC7949476; doi:10.1136/jitc-2020-001946)
Supplement: Supplementary data [file jitc-2020-001946supp003.pdf]

Supplementary Methods

Vectors and cell transfections

shRNA-STAT3 and its negative control for (mU6-MCS-Ubi-Luc) lentiviral vectors were purchased from Shanghai GeneChem Co. mU6-MCS-Ubi-Luc-shRNA-STAT3 was transfected into three ICC cells: HuCCT1, RBE and SG231. mU6-MCS-Ubi-Luc lentiviral vectors were used as controls. Stably transfected clones were validated by immunoblotting.

RNA isolation, qRT-PCR and RT^2 profiler PCR array

We extracted total RNA from cells using Trizol reagent (Invitrogen, Carlsbad, CA) in accordance with the manufacturer's instructions. We measured mRNA expression via qRT-PCR using an ABI7900HT instrument (Applied Biosystems, Foster City, CA). Quantitative RT-PCR was done using a SYBR PrimeScript RT-PCR Kit (Takara Bio, Shiga, Japan) in accordance with the manufacturer's instructions. We used GAPDH as an internal control. We calculated relative mRNA levels based on the Ct values and normalized using GAPDH expression, according to the equation: $2^{-\Delta Ct}$ [ΔCt = Ct (target gene) - Ct (GAPDH)].

RT^2 profiler PCR array was performed for screening TANs and TAMs-derived cytokines/chemokines by using the Human Cytokines & Chemokines PCR Array (catalogue number: PAHS-150Z, SABiosciences, Hilden, Germany). The RT^2 Profiler array was probed by using the Profiler PCR Array System and SYBR Green/Fluorescein qPCR Master Mix (SABiosciences) in an ABI 7900 sequence analyzer (Applied Biosystems, Carlsbad, CA,USA) in accordance with the manufacturer's protocol. Gene

expression was analyzed using the dedicated Web-based software package (http://www.superarray.com/pcr/arrayanalysis.php), which automatically performs all $2^{-\Delta Ct}$ based fold-change calculations from the specific uploaded raw threshold cycle data.

Western blot

Western blotting was performed as previously described [1]. Briefly, we generated total cell lysates, and proteins were separated on 10% SDS-PAGE, and then transferred the proteins to polyvinylidene difluoride (PVDF) membranes. The membranes were washed and blocked. Primary antibodies were applied, followed by horseradish-peroxidase–conjugated secondary antibodies. Antibody binding was detected by enhanced chemiluminescence assays.

Enzyme-linked immunosorbent assay (ELISA)

We determined the level of cytokines/chemokines in cell culture supernatants by using the corresponding quantikine human ELISA kit (R&D Systems) in accordance with the manufacturer’s instructions. Briefly, we added 100 μL of sample to each well and incubated the plates for 2.5 h at RT. The plates were washed and incubated with the conjugate for 2 h. After washing, we determined immunoreactivity by adding substrate solution, and the absorbance was determined using a Microplate Spectrophotometer (Bio-Rad). A curve of the absorbance versus the concentrations of cytokines/chemokines in the standard wells was plotted.

TMA and immunohistochemistry

TMAs were constructed as previously described [2]. We took two 2-mm diameter core biopsies from the donor blocks and transferred these to the recipient paraffin block at

predefined array positions. We constructed TMA blocks including 359 cases in cohort 2.

Immunohistochemical staining was performed by the avidin-biotin-peroxidase complex method. Briefly, after rehydration and microwave antigen retrieval, primary antibodies were applied to slides, incubated at 4°C overnight, and followed with secondary antibody incubation (GK500705, Gene Tech, China) at 37°C for 30 min. Staining was carried out with DAB and counter-staining was performed with Mayer’s hematoxylin. In all assays, we included negative control slides with the primary antibodies omitted.

Evaluation of immunohistochemical variables

Immunohistochemical staining was assessed by three independent investigators who were blinded to patient characteristics, and discrepancies were resolved by consensus. Under 200× magnification, photographs of three representative fields were captured by the Leica QWin Plus v3 software; identical settings were used for each photograph. For the CD66b and CD68 staining in TMAs, the number of positive cells was calculated in each 2-mm-diameter cylinder and expressed as the mean value of the triplicates (cells/spot) as described previously [3]. Median values were used as a cut-off in subsequent analyses unless specified. STAT3 density in TMA was determined using Image-Pro Plus v6.2 software (Media Cybernetics, Inc., Bethesda, MD). Integrated optical density of all positive STAT3 staining in each photograph was measured and its ratio to the total area of each photograph was calculated as the STAT3 density. The median STAT3 density was determined using immunohistochemistry and used as the cut-off in subsequent analyses.

Cell proliferation, matrigel invasion and colony formation assays

ICC cells were seeded in 100μL of media in a 96-well plate (2000 cells/well), and 10 μL CCK-8 solution (Dojindo) was added to the cells at the indicated time points. The cells were then incubated for an additional 2 h. The number of viable cells was determined by absorbance measurements (450 nm).

To assess cell invasion, we used 24-well Transwell plates 8-μm pores (Minipore), which were pre-coated with Matrigel (BD Biosciences). The lower chamber contained 600 μL Dulbecco's modified Eagle medium (DMEM) with 10% FBS. Cells were added to the upper chamber (1×10^5 cells) in 100 μL media supplemented with 1% FBS. After 48h, we removed the remaining cells and Matrigel in the upper chamber. Cells that had invaded the lower membrane surface were fixed with 4% paraformaldehyde and stained with Giemsa. Cells from six microscopic (200×) fields were counted.

To assess the colony formation abilities of these cells, 500-1000 cells were seeded into each well of 6-well plates and incubated at 37°C for 12-16 d. Cells were then fixed with 100% methanol before staining with 0.1% crystal violet. Image-Pro Plus v6.2 (Media Cybernetics) was used to count the megascopic cell colonies.

***In vivo* assays for tumor growth and metastasis**

For mouse ICC models, 1×10^7 HuCCT1, RBE and SG231 cells transfected with or without shRNA-STAT3 alone, or these cells were co-injected with TANs and/or TAMs purified from tumor tissues of ICC patients into the subcutaneous space of the upper left flank region of NOD-*Prkdc*^{scid} *IL2rg*^{tm1}/Bcgen mice. Cell suspensions consisted of a 1:10 ratio of the cell mixture (TANs : ICC cells, TAMs : ICC cells or TANs and TAMs : ICC cells) and in these groups, TANs and TAMs were injected into the tumor at the indicated dosage

biweekly for 3 consecutive weeks from day 14 after inoculation. In some experiment groups, mice were given STAT3 inhibitor, S3I-201, intraperitoneally at 10 mg/kg every other day for 3 consecutive weeks from day 14 after inoculation. All mice were monitored once every 5 days and killed 5 weeks later. The volume of tumors was calculated in cm^3 as follows: $V = ab^2/2$ (with a and b representing the largest and smallest tumor diameters) [4]. Upon sacrifice, the tumours were recovered and the volume of each tumour was further determined. Lungs were removed and embedded in paraffin and the total number of lung metastases was counted under the microscope as described previously [5]. The metastases were classified into four grades on the basis of the number of tumor cells present at the maximal section for each metastatic lesion: grade I, ≤20 tumor cells; grade II, 20-50 tumor cells; grade III, 50-100 tumor cells; and grade IV, >100 tumor cells.

References:

1. Zhou S, Tan C, Dai Z, et al. Tacrolimus enhances the invasion potential of hepatocellular carcinoma cells and promotes lymphatic metastasis in a rat model of hepatocellular carcinoma: involvement of vascular endothelial growth factor-C. *Transplant Proc* 2011;43:2747-54.
2. Gao Q, Qiu SJ, Fan J, et al. Intratumoral balance of regulatory and cytotoxic T cells is associated with prognosis of hepatocellular carcinoma after resection. *J Clin Oncol* 2007;25:2586-93.
3. Li YW, Qiu SJ, Fan J, et al. Intratumoral neutrophils: a poor prognostic factor for hepatocellular carcinoma following resection. *J Hepatol* 2011;54:497-505.
4. Wang L, Tang ZY, Qin LX, et al. High-dose and long-term therapy with interferon-alfa inhibits tumor growth and recurrence in nude mice bearing human hepatocellular carcinoma xenografts with high metastatic potential. *Hepatology* 2000;32:43-8.
5. Tian J, Tang ZY, Ye SL, et al. New human hepatocellular carcinoma (HCC) cell line with highly metastatic potential (MHCC97) and its expressions of the factors associated with metastasis. *Br J Cancer* 1999;81:814-21.
